# Northern Ohio Dental Society

**Published:** 1873-08

**Authors:** C. B. Knowlton

**Affiliations:** Put-in Bay Island


### NORTHERN OHIO DENTAL SOCIETY.

PUT-IN BAY ISLAND.
June 10th, 1873. 10 a. m.

Meeting called to order by the President, Dr. Robinson Minutes of the last meeting read and approved.

Members present, D. R. Jennings, L. Buffett, B. Strickland, B. F. Robinson, W. P. Horton, R. R. Peebles, J. Stephan, Cleveland; F. S. Whitslar, Youngstown; E. J. Waye, H. M Reid, C. T. Strond, Sandusky; C. B. Knowlton, Oberlin; A. F. Price, Fremont.

Treasurer's report received and referred to Auditing Committee. Dr. Waye was appointed Auditing Committee.

Bill of Corresponding Secretary referred to the Auditing Committee.

Adjourned to 2 p. m.

Report of Auditing Committee received and acce [illegible]ed,

Examining Committee reported and recommended the names of J. E. Phelps, of Chagrin Falls, to become an active member, and H. A. Smith, of Cincinnati, an honorary member of this Association, both of whom were elected. On motion, Dr. E. M. Smith, of Jackson Michigan, and A. B. Robinson, of Butler County, became honorary members of the association.

The first subject for discussion was the relative value of the cheaper materials for filling teeth.

The subject was introduced by an essay by Dr. Waye who spoke of the materials used, classed under the name of cheap materials. Considered tin superior to all others when used with the same amount of skill that gold was; did not consider any of the preparations of amalgam fit material for filling, as he had not found any but what would contract more or less, leaving walls of cavity exposed, thereby destroying all the purposes for which it was used.

Hill's Stopping, the oxy-chloride of zinc, and the various preparations of gutta-percha, are useful in their place; but it must depend upon the judgment of the operator as to where and when they are to be used.

The essay was highly creditable to its author. Motion carried that essay be received.

Dr. Harroun had had a good deal of experience in the use of tin fillings, which he considers next to gold; thought tin required as much skill and time to make a perfect filling as gold; could not recommend amalgam: for temporary fillings would use Hill's Stopping.

Used oxy chloride of zinc in a good many cases as a capping to exposed nerves, first applying a coating of collodion; had been successful in nearly every case in applying the collodinn; would use thick applied on a piece of spunk, dried by using a hot air syringe; did not think the collodion would contract when not applied in excessive quantities.

Dr. Strickland preferred a solution of gutta-percha dissolved in chloroform as a capping directly over the nerve, as he had some difficulty with collodion, on account of its contracting.

I think in the use of amalgam, a great deal depends upon the manner of its preparation; I would use but little mercury, preferred it in some cases to tin; in using amalgam would grind and wash thoroughly, and press out all the mercury that was possible; much better results would be obtained from amalgam if more pains were taken in its manipulation.

Dr. Smith thought it necessary that there should be some material which could be used with less expense than gold, I have not been able as yet to find any material that would compare with gold; did not use amalgam as extensively as formerly.

Dr. L. Buffett thought amalgam was used more or less by nearly all dentists; very few were so situated that all of their patients could pay for gold; regarded tin as the next best filling to gold, and that it could be used in most of cases; next to tin would take Hill's Stopping; had not been successful in the use of oxy-chloride as a capping to nerves.

Dr. J. E. Robinson thought we ought to all consider how we can do the most good in our profession, did not think we ought to entirely discard the use of amalgam; fully agreed with essayist in regard to the use of tin; thinks a great many teeth have been saved by amalgam fillings in the mouths of patients who were not able to pay for gold; would sooner save a tooth as long as possible with amalgam, than extract it and put in a poor substitute.

Dr. Horton thought there was but a small proportion of dentists who could get along without the use of some of the cheaper materials for filling teeth; that the value of amalgam depended largely upon the manner of its preparation and introduction into cavity; had seen amalgam fillings when properly prepared last fifteen to twenty years; there were cases where amalgam was to be prefered to tin; as amalgam fillings were ordinarily put in, four out of five would fail, owing more to the manner of its manipulation than to the material.

In children's teeth often used tin; thought in the six year molar, that the excessive mercury from amalgam fillings would cause destruction of the nerve. There was some good in all of the cheap materials and it must depend upon the good judgment of the dentist when and how they should be used.

Dr. Smith doubted if an amalgam filling would destroy the pulps sooner certainly than a filling of any other metal.

Dr. Strickland thinks if there was no mercury to evaporate that the fillings would not contract. In putting in amalgam fillings would pack carefully against the walls of the tooth; would take two or three thicknesses of tin foil and burnish against the filling to absorb all the mercury possible; have the patient call in a day or two and have the filling polished so as to get a perfectly smooth surface; had seen such fillings retain their polish and preserve the teeth a great many years; did not want to be understood as claiming that it would always secure the best results; thought we ought to investigate the kind and preparation of material used in the different amalgams.

Dr. Stephan remarked, that the space around large amalgam fillings, was not caused so much by the evaporation of mercury, as by the edges of the amalgam breaking off—it being a very brittle substance.

Dr. Reid used some amalgam in his practice, though not as much as formerly, but used more gold, as he had been able to educate his patients to a higher standard of dentistry. In a well-prepared amalgam filling one-sixth of its weight is mercury—in a large proportion of amalgam fillings one-fifth is mercury.

He related a case where the bicuspid teeth were so badly decayed that there was not sufficient strength to fill with gold; filled roots and crowns with oxy-chloride of zinc; cut away from edge of cavity so that more of it was exposed to surface, then filled with amalgam; as yet had seen no discoloration of the teeth.

Dr. Peebles thinks amalgam fillings are brought largely into discredit from the fact that a great many teeth are filled with amalgam, that are not considered worth filling with any other material.

Dr. E. W. Smith thought that the discolored bone around amalgam fillings was seldom found in a softened condition, but it was harder than the natural condition.

Dr. Knapp believes that if gold had been stuffed in teeth in the same careless manner in which the most of amalgam fillings are, that the failure would be as great as with amalgam.

Dr. Knowlton thought that one of the main difficulties in amalgam fillings is that it was used more in a class of teeth where the pulps were destroyed so that there was less vitality in the tooth, and more liability to break down.

Adjourned to half-past seven.

EVENING SESSION.

Dr. Palmer considered tin foil next to gold; never was an advocate of amalgam; thought amalgams had been much improved in the last few years by using pure material; could use tin foil where amalgam could be used; would use Guilloi's Cement in preference to any other material for a cheap filling. It will become harder than any of the other preparations of oxy-chloride of zinc. As a capping for exposed pulps, it was to be preferred, as it caused less pain than any of the other preparations of zinc; would use carbolic acid before putting in the cement.

Dr. Spelman does not think that the use of any escharotic to an exposed nerve, is a good plan; does not like to use any material that causes pain and irritation to the pulp; in a large majority of cases it will cause destruction of the pulp; the treatment should be by using some material that would be soothing, and not irritating to it; would use a solution of phenol covered with an arnica plaster dried by a hot air syringe, and then use the oxy-chloride of zinc; believes that chloride of zinc destroys the carbolate of albumen which is formed by h. application of carbolic acid.

Dr. Palmer in filling with tin does not use the mallet; thought it did not require the sam [illegible] of pressure that gold does.

The Second Subject for discussion was then taken up: "Neuralgia and Pain of the Dental Tissue and Treatment."

Dr. Whitslar: We are often at a loss to give the proper cause of neuralgia, and no one ought to be better able to do so than the Dentist, though a large share of the people, will first call upon their physician foi treatment. It is necessary for the dentist to have a thorough knowlege of anatomy and physiology, in order to have a proper understanding of the treatment. Related a case facial neuralgia cured by using hypodermic injection of morphine; urged upon the profession the necessity of being thoroughly posted so as to have an intelligent understanding of the different parts of the human system.

Dr. Strickland would inquire what is neuralgia. His understanding of neuralgia was that it is a disease of the only portion ofthe system which gave pain, which was the nerves. Anything which irritated the nerves, would cause pain in some part of the body. Whenever any of the nerves of head or face are irritated and cause pain, we generally think that the cause was in the teeth, when perhaps the true cause was very remote from them, so that it was not always safe to trust to where the patient located the pain. Often local application will give temporary relief, but a permanent cure generally depends upon constitutional treatment, and the assistance of nature.

Dr. Horton considered neuralgia a disease which required more study than a large class of dentists were disposed to give it—one which required a great deal of thought. The most eminent physicians and skillful surgeons are often at a loss to give the cause and treatment of facial neuralgia ; had made local application of morphine in some cases, which gave relief ; apprehended that the same was generally owing to a weakened condition of the patient, who would require constitutional treatment and a building up of the system generally, before any permanent cure could be effected.

Dr. Spelman wished to give his theory why the dentine of a tooth was less sensitive after the rubber dam was applied than before. First, he thought that the pressure of the dam upon the tooth tended to raise it in its socket, partially paralyzing the nerve at the end of root. Second, when the cavity is moist there is a current of electricity from the body of the operator to the tooth ; but if the tooth is keep perfectly dry it becomes as much a non-conductor as so much ivory.

Dr. Buffett thinks a tooth cannot be so perfectly isolated as to become as much a non-conductor as a piece of ivory.

Dr. Hildreth does not think that the passage of electricity was painful to the patient.

Adjourned to 9 A. M. to-morrow.

The subject of the evening's discussion was then resumed.

Dr. Harroun—The principal part of neuralgic pains comes from a diseased condition of the teeth, either a diseased pulp, periosteal inflammation, crowded condition of the teeth, and other causes. When the patient was inclined to be bilious, he treated by giving cathartics and following with tonics.

Dr. Waye—The principal neuralgia that dentists have to treat is facial neuralgia. Physicians often treat neuralgia of face without examining the teeth, when there are but few cases of facial neuralgia but are influenced by the teeth—the dentist is the proper person to treat such cases.

Dr. Peebles cured a case of neuralgic pains in spine by extracting lower wisdom teeth and prescribing tonics.

Dr. Hildreth thought neuralgia was but little understood by either dentists or physicians. Neuralgia means pain in nerve. To treat neuralgia we should be able to form a correct diagnosis of case. We frequently have neuralgia caused by malaria. In bilious persons would prescribe cathartics in some cases of periodical neuralgia; had prescribed tonics with very happy effect. The supra orbital branch of the fifth pair of nerves is frequently the seat of neuralgic pains, and has to be treated upon general principles, and sometimes requires the most heroic treatment; had sometimes prescribed opium in large doses. A large proportion of the causes of facial neuralgia are by the wisdom teeth; the only radical cure in those cases was to remove the tooth. He related a case where a patient had a severe pain in the arm relieved by extracting the root of the tooth affected with exostosis.

Motion made and carried that we proceed to election of officers for ensuing year, which resulted as follows.

President—Dr. S. P. Hildreth; Vice Pres.—D. R. Jennings; Recording Secretary—C. B. Knowlton; Corresponding Secretary—Charles Buffet: Treasurer—J. E. Robinson; all of whom were elected by acclamation.

Dr. Whitslar and Dr. Harroun conducted the newly-elected President to the chair.

Dr. Hildreth thanked the association for the compliment conferred upon him.

On motion, the retiring address of Dr. B. F. Robinson, was listened to with great interest.

On motion, Dr. Palmer had permission to exhibit and explain some very fine instruments.

The following gentlemen were elected delegates to the American Dental Association; E. J. Waye, R. R. Peebles, J. E. Robinson, F. S. Whitslar, L. Buffett, B. Strickland, H. M. Reid.

On motion, the following essayists were appointed for the next annual meeting; Drs. Horton, Waye, Buffett, Harroun, Strickland, Palmer, Whitslar.

On motion, adjourned to 2 p. m.

AFTERNOON SESSION, 2ND DAY, 2 P. M.

Meeting called to order by the president, Dr. Hildreth.

On motion, the Association adjourned to meet at 8 p. m.

EVENING SESSION, 2ND DAY.

Meeting called to order by the president, Dr. Hildreth.

Dr. L. C. Kelsey being recommended for membership in this Association by the board, was duly elected to the same. Discussion being in order the following subject was taken up: "Constitutional Diathesis," and opened by the reading of a paper by Dr. Hawxhurst, treating of sympathy and action of organs and functions as connected with diathesis.

Dr. Taft: All the conditions must be known fully by the man who successfully treats disease, and to this end a knowledge of foundation principles—anatomy, pathology, and physiology, which he elaborated—showing the most intimate dependance of one organ upon another, their sympathetic relations, etc.

Dr. Spelman inquired of Dr. Taft what he understood by the term diathesis? he understanding it to mean a peculiar condition of parental heritage.

Dr Taft understands diathesis to mean a predisposition or a condition tending to diseased action. It may be general or local as applied to a particular organ or function. Predisposition may, and often does extend through successive generations, sometimes missing and then striking in again.

Dr. Hawxhurst: It would be interesting to know the difference between temperament and diathesis. The Dr. spoke at some length showing that temperament and diathesis are synonymous.

Dr. H. A. Smith and others continued the discussion.

On motion, discussion on topics closed.

On motion, it was resolved that the chair appoint an executive committee, who appointed the following gentlemen; B. Strickland, E. J. Waye, Chas. Buffett.

On motion, the Association adjourned, to meet at Put-in Bay on the second Tuesday of June, 1874.

C. B. Knowlton.

*Sec'y.*

